# Deciphering the impact of cerebrospinal fluid on stem cell fate as a new mechanism to enhance clinical therapy development

**DOI:** 10.3389/fnins.2023.1332751

**Published:** 2024-01-12

**Authors:** Klaudia Radoszkiewicz, Aleksandra Bzinkowska, Magdalena Chodkowska, Paulina Rybkowska, Monika Sypecka, Ilona Zembrzuska-Kaska, Anna Sarnowska

**Affiliations:** Translational Platform for Regenerative Medicine, Mossakowski Medical Research Institute, Polish Academy of Sciences, Warsaw, Poland

**Keywords:** cerebrospinal fluid, artificial cerebrospinal fluid, embryonic cerebrospinal fluid, adult cerebrospinal fluid, neural stem cell, cell therapy, neurological disorders

## Abstract

Neural stem cells (NSCs) hold a very significant promise as candidates for cell therapy due to their robust neuroprotective and regenerative properties. Preclinical studies using NSCs have shown enough encouraging results to perform deeper investigations into more potential clinical applications. Nevertheless, our knowledge regarding neurogenesis and its underlying mechanisms remains incomplete. To understand them better, it seems necessary to characterize all components of neural stem cell niche and discover their role in physiology and pathology. Using NSCs *in vivo* brings challenges including limited cell survival and still inadequate integration within host tissue. Identifying overlooked factors that might influence these outcomes becomes pivotal. In this review, we take a deeper examination of the influence of a fundamental element that is present in the brain, the cerebrospinal fluid (CSF), which still remains relatively unexplored. Its role in neurogenesis could be instrumental to help find novel therapeutic solutions for neurological disorders, eventually advancing our knowledge on central nervous system (CNS) regeneration and repair.

## 1 Neural stem cells for treating neurological disorders – current challenges

Neural stem cells (NSCs) are known as the perfect candidates for cell therapy because of their rich neuroprotective and pro-regenerative properties. The promising outcome of neural stem cell-based preclinical studies in neurological diseases encourages researchers to further investigate their properties and responses, and move them to the clinical level. However, our knowledge regarding neurogenesis and the mechanisms accompanying this process is still limited. Due to the complexity of the central nervous system (CNS), neurological disorders still lack effective clinical outcomes, thus, there is a need to find a more successful therapeutic approach and target the factors which are involved in their pathogenesis. Currently, these cells cannot only be isolated from different regions of the fetal or adult brain and spinal cord but can also be generated from other cell types, including embryonic stem cells (ESCs), induced pluripotent stem cells (iPSCs) and reprogrammed somatic cells (induced neural stem cells-iNSCs). In addition, neural-like cells can be obtained from mesenchymal stem/stromal cells (MSCs) (Tang et al., 2017; Willis et al., 2020). Such a wide variety of cell sources, by enriching the availability of NSCs, seems to provide safer and easier ways for preparing *in vitro* models of neurological diseases or using them for *in vivo* treatment but is their effect/response the same as for the native NSCs? What conditions would be able to mimic the natural brain environment? Despite several pros of NSC application *in vivo*, researchers are aware of many cons, including poor cell survival, poor integration into the host tissue as well as uncontrolled differentiation (Mothe et al., 2013; Wu et al., 2018). We wondered if there could be any particular factor that could have been overlooked. Although NSCs and neural niche components, including various cell types and matrix factors, are being extensively studied, there is limited data pertaining to the impact of the fundamental fluid on NSC which is present in the brain. Thus, here, we want to sum up our current knowledge in that aspect. It has been suggested previously that cerebrospinal fluid (CSF) plays a vital role not only in brain development, but also in neuroectodermal stem cells’ survival, proliferation, and differentiation processes, thus, we find it a perfect model to study NSC behavior. In this review, we analyze the current knowledge on CSF’s influence on stem cell fate *in vitro*, with the hope of finding out what still needs to be further investigated in order to better understand the processes that occur in the brain.

## 2 Cerebrospinal fluid – a conduit to further improve NSC therapy development

Cerebrospinal fluid (CSF) is a colorless liquid produced by the filtration of blood in the choroid plexus (ChP), which is an epithelial barrier that prevents free entry of toxic molecules or drugs in the circulation from reaching the brain. Together with the blood–brain barrier (BBB), the blood-spinal cord barrier (BSCB), and the blood-CSF barrier (B-CSF-B) make up the CNS barrier (Stoop et al., 2010). Humans produce approximately 500 mL of CSF a day and the total volume of CSF at a given time is usually about 150 mL (Spector et al., 2015). To obtain human CSF, the lumbar atraumatic needle is usually placed at the level of L3-L4 vertebrae, so that the introducing needle enters below the level at which the spinal cord ended. 10 to 15 mL of cerebrospinal fluid is collected into the polystyrene probes, centrifuged (2000 *g*, 10 min, RT) and biobank in-80C for further analysis (Liu and Duff, 2008). The methods used in animal models differ depending on the available instruments, animal model, and study group. Most often the animal is placed in the head-first prone position with the head remaining lowered due to approximation of the nasal clamp of the stereotactic frame at the head, just above the eyes. The hair around the cavity of the cisterna is shaved. Then the gingival needle connected to the microsyringe by a very thin and flexible tube or Hamilton syringe with needle or capillary is used. Once the dura over the cisterna magna is exposed, a needle or capillary is used to gently pierce it. Then CSF in amount of 40–70 μL is collected and frozen (Pegg et al., 2010; Lim et al., 2018).

### 2.1 CSF composition

**Figure 1:**
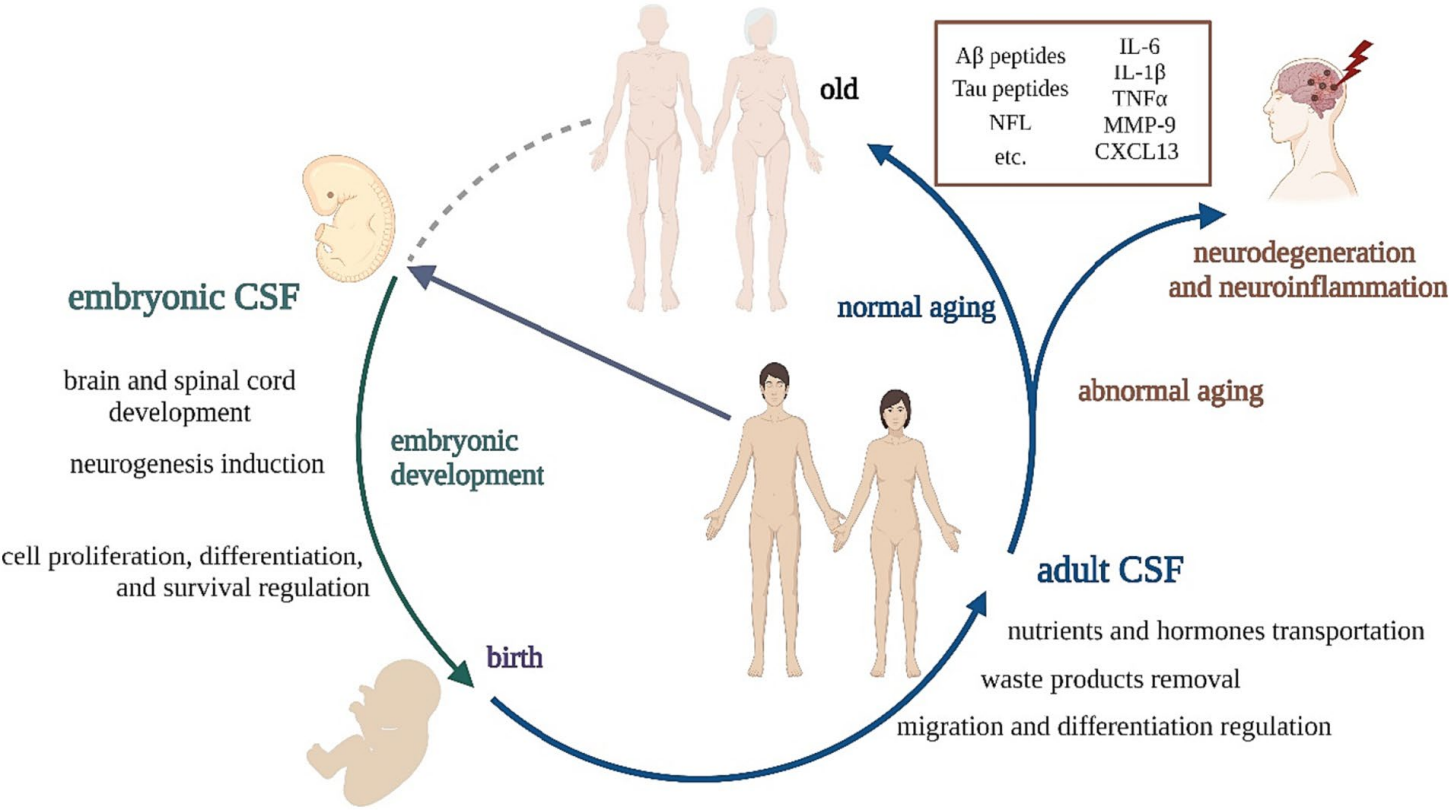
The scheme presenting the role of CSF at particular life stages.

Circulating CSF ensures homeostasis in the brain – it maintains metabolic clearance and also protects the CNS from mechanical shocks. The composition of CSF has been deeply investigated over the years. CSF contains ions including Na^+^, Cl^−^, HCO3^−^, K^+^, Ca^++^, Mg^++^, Mn^++^; vitamins (e.g., Vitamin C, thiamine monophosphate, pyridoxal phosphate) and a great number of different proteins (Schilde et al., 2018). It is assumed that 80% of these proteins originate from blood and that the remaining 20% is released from the neural tissue. CSF of healthy people contains less than five cells per μL (Olsson et al., 2016). Due to its close proximity to the brain, CSF plays a crucial role when it comes to the diagnosis of various neurological disorders – the composition of CSF reflects biological processes that take place in the CNS. For example, Lepennetier and co-workers observed increased levels of multiple cytokines in CSF obtained from patients with neuroinflammatory diseases when compared to patients with non-inflammatory neurological diseases (Lepennetier et al., 2019). Thus, many studies concentrate on finding biomarkers characteristic of particular disorders that can be found in circulating CSF (Figure 1) (Olsson et al., 2016). Analyzing single-cell transcriptomics in CSF has revealed immune responses across a spectrum of neurological disorders, from inflammatory and degenerative to infectious and oncological CNS conditions. Despite the current lack of large CSF datasets, establishing a robust reference atlas demands collaborative efforts among multiple centers. Essential steps include optimizing CSF cell preservation, integrating existing datasets, and ensuring the resulting annotated datasets are publicly accessible, featuring interactive visualization (Heming et al., 2022). Relatively recent scientific evidence points to the role of CSF in regulating the sleep–wake cycle through its effect on prostaglandin synthesis, specifically prostaglandin D2 (Hayaishi, 2000). Moreover, different cells throughout the body, including cells making up the central nervous system (CNS), secrete extracellular vesicles (EVs). EVs are involved in intercellular communication via the transfer of numerous membrane receptors, proteins, lipids, RNA, and miRNA between neighbor and more distant cells (Jarmalavičiūtė and Pivoriūnas, 2016). EVs are secreted by neurons and glial cells into the CSF which remains in direct contact with the CNS. EVs play a critical role in bidirectional crosstalk between the CNS and the periphery, as they are able to cross the blood–brain barrier (BBB) (Honorato-Mauer et al., 2023; Kong et al., 2023). As EVs can cross the BBB and later circulate in the CSF, their contents can serve as biomarkers of the pathological processes that might be happening in the brain. One of the most promising biomarkers of different neurological disorders is miRNAs, which have been identified as the cargo of EVs (Mori et al., 2019; Šalamon Arčan et al., 2023).

Though CSF is an important diagnostic tool, its components and their role are one of the most under-explored areas of neuroscience. Previously, CSF was considered to be a fluid with basic physiological and mechanical functions. Nowadays, studies show that CSF plays a critical role in complicated brain physiology, especially during development, modulating the functions of neural stem cells (NSC) (Wichmann et al., 2022) and brain restoration.

### 2.2 Embryonic CSF (eCSF) vs. adult CSF (aCSF)

Depending on the stage of development, CSF changes its composition and function. Embryonic CSF (eCSF) produced during embryogenesis is essential for proper development of the brain and spinal cord. It contains growth factors, cytokines, and other signaling molecules that regulate cell proliferation, differentiation, and survival in the developing nervous system. eCSF is also important for regulating the size and shape of the developing brain ventricles (Gato et al., 2014; Bueno and Garcia-Fernàndez, 2016; Chang et al., 2016).

The eCSF can support the fate of NSCs at any stage of their development, including the expansion of the undifferentiated NSC population or by inducing neuronal differentiation, migration, and final neuronal maturation (Alonso et al., 2017). Direct contact between eCSF and NSCs is necessary for their survival, replication, and neural differentiation and is considered a major source of signals in NSCs’ niche regulation (Gato et al., 2005). Nevertheless, these inducive properties change suddenly during adulthood, where CSF has been described as NSCs’ ‘migratory guidance.’ aCSF has mitogenic properties but has lost its ability to induce neurogenesis. Some studies have associated ontogenic changes in the composition of eCSF and aCSF with a dramatic reduction of the neurogenic potential of the adult brain (Alonso and Gato, 2018).

Due to the existence of technical and ethical constraints, no studies of eCSF have been conducted in humans to date. As it is crucial to learn more about the impact of eCSF on NSCs’ neurogenesis, this matter might be studied with the use of animal models, especially mammals. This may pave the way for the preparation of art-CSF that could reflect the neurogenic potential of native CSF and therefore unlock the possibility of enhancing neuroregeneration in humans.

On the other hand, Pellegrini and her group created vascular plexus (ChP) organoids that reproduced key morphological and functional features of the human vascular plexus. This model allows the created ChP organoids to secrete a CSF-like fluid highly similar to *in vivo* CSF. Moreover, these organoids and CSF-like fluid also mature over time, reaching a state highly similar to postnatal stages and adulthood (Pellegrini et al., 2020).

Adult CSF (aCSF) is produced and circulated in the brain and spinal cord throughout adulthood. Its primary functions include cushioning the brain and spinal cord, removing waste products, and transporting nutrients and hormones. eCSF and aCSF differ considering their composition and function. Studies show that human eCSF has a higher concentration of total proteins than aCSF (Gato et al., 2005; Parada et al., 2005a) and a similar situation can be observed in other mammals, for example, in rat and sheep embryos. On the other hand, significant phylogenetic differences in CSF’s maturation have also been identified, possibly reflecting the particularities of CNS development across species (Bueno et al., 2020).

### 2.3 CSF in-laboratory-models

These differences are useful to study the neurogenesis-related aspects. The type of CSF used may prove to be a key factor in potential cell therapies in the central nervous system.

Moreover, there are CSF in-laboratory-models that closely mimic the composition of CSF. They can be made using a variety of techniques, including mixing various biochemical components in specific proportions, culturing cells in media that contain CSF, or using specialized microfluidic devices to create artificial CSF-like (art-CSF) environments.

One of the most advanced models in brain research seems to be the microfluid system. These systems can be designed to mimic the complex microenvironment of the brain, allowing for the study of the transport and diffusion of molecules within the CSF and their effects on brain cells. Furthermore, microfluidic systems can also be used to study the interactions between CSF and brain cells, such as neurons and glial cells (Cliver et al., 2021). In order to closer mimic the brain environment, Cho and his group developed a brain-mimetic 3D organoid culture system by combining two basic elements: human brain tissue-derived ECM and a microfluidic chamber device.

CSF models in the *in vitro* research might help to understand the underlying mechanisms of brain function and disease, and may ultimately lead to the development of new therapeutic interventions for neurological disorders (Table 1).

**Table 1:** The comparison of embryonic cerebrospinal fluid (eCSF) and adult cerebrospinal fluid (aCSF).

	eCSF	aCSF
Stage of development	Before the formation of the choroid plexus	After the formation of the choroid plexus
Main functions	Neurogenesis induction; brain and spinal cord development	Waste products removal; nutrients and hormones transportation
CSF composition	Rich in protein – high eCSF concentration of proteins such as albumin, fetuin, alpha-fetoprotein transferrin, and lipoproteins	Lack of protein (the protein concentration after birth falls dramatically)

## 3 Impact of CSF on stem cell fate

### 3.1 Effect on native NSCs

As mentioned before, NSCs transplantation is a promising tool for stem cell therapy development what is especially important for neurological diseases in which endogenous NSCs are not able to supply enough cells to repair the injured neural tissue (Arvidsson et al., 2002; Pluchino et al., 2008; Willis et al., 2020). These cells are self-renewing and multipotent, capable of differentiating into three major cell types of CNS, astrocytes, oligodendrocytes, and neurons, which makes them a perfect candidate for neurodegenerative disorders therapy. During embryonic development, they give rise to neurons and glia, while in adulthood, they are found mostly within the subventricular zone (SVZ) and in the subgranular zone (SGZ) of the hippocampus. Through the last two decades, researchers have found that CSF factors impact NSC activity, playing a significant role both in physiological and pathological processes in the brain with adult neurogenesis included (Alcázar et al., 1998; Redzic et al., 2005; Lehtinen et al., 2011; Silva-Vargas et al., 2016).

The study findings regarding the role of CSF on NSCs seem to be inconsistent. In the study of Ma and coworkers, it has been observed that human aCSF did not support the survival of newborn rats’ neurons. Moreover, it inhibited neurogenesis. Similarly, NSCs derived from rat fetuses, when exposed to human aCSF, differentiated only into glial cells (Ma Y. et al., 2013). These results suggest a potential inhibitory effect of human CSF on neuronal development and a bias toward glial cell differentiation. However, in a study of another group performed on HTX rats, it was revealed that rat aCSF (obtained from newborn rats) stimulated neurosphere differentiation into neurons, glial cells and ependymal cells as well (Henzi et al., 2018). These results suggest that specific experimental conditions including different species origin of CSF may strongly contribute to its final effect on presented cells.

The effect of CSF derived from patients with neurological diseases was also investigated. Buddensiek’s group reported that adult human leptomeningeal CSF promotes survival, and astrogliogenesis of fetal NSCs obtained from rats (Buddensiek et al., 2009). They have continuously observed such effects on human NSCs. Moreover, such cell culture conditions lead to stronger cell extension outgrowth and loss of cell proliferation potential measured with Ki67 and BrdU (Buddensiek et al., 2010). These results seem to provide evidence for the presence of factors regulating NSC proliferation and differentiation in adult CSF. The authors proposed that the CSF factors that presumably influenced NSC differentiation could be bone morphogenetic proteins (BMPs), such as BMP7 or BMP4. The first one is expressed in the choroid plexus and secreted into the CSF. It was shown to inhibit the CSF-induced neuronal dendritic outgrowth, while the second one promoted the differentiation via ERK pathway activation and GSK3β inhibition (Dattatreyamurty et al., 2001; Moon et al., 2009; Buddensiek et al., 2010). In addition, BMP2, BMP3, BMP4, BMP5 and BMP6 are also expressed in the choroid plexus but it is not known if they are present in CSF (Jensen et al., 2021). It has been observed by Zveik and his group that CSF from patients with relapsing multiple sclerosis (rMS) and progressive MS (pMS) enhance the ability of mice oligodendrocyte progenitor cells (OPCs) to differentiate into mature oligodendrocytes and to express immune functions. OPCs exposed to CSF from rMS patients were more morphologically mature compared to cultures exposed to CSF from pMS. After MS-patients-derived CSF exposure, OPCs become immune activated by NFκB activation, processing and presenting antigens to T cells, and secreting anti-or pro-inflammatory cytokines (Zveik et al., 2022). It is known that in case of a brain damage, the injured brain releases specific factors to stimulate endogenous neurogenesis and these factors might appear in CSF. Another study examined the relationship between neurogenesis and subarachnoid hemorrhage (SAH) features using CSF. The group observed that CSF from SAH patients enhanced the proliferation capacity of cultured rat NSCs (Chen et al., 2018). They showed that the capacity to proliferate of these cells is correlated with the severity and functional outcome of the disease. Another explanation for these observations may be the presence of blood in CSF obtained from patients with SAH. The presence of platelets (one of the main morphotic components of the blood) and their subsequent degranulation results in the release of trophic factors such as platelet-derived growth factor (PDGF), epithelial growth factor (EGF), vascular endothelial growth factor (VEGF), endothelial cell growth factor (ECGF), fibroblast growth factor (FGF), transforming growth factor beta (TGFβ) and insulin-like growth factor (IGF). Which affect stem cell behavior by inducing proliferation and differentiation (Masoudi et al., 2016).

Overall, the role of aCSF appears to involve providing a supportive microenvironment for NSCs, promoting their survival, influencing their differentiation into specific cell types, and potentially containing factors like BMPs that modulate NSC behavior.

It has been also examined whether using CSF obtained from different developmental stages would impact the NSC culture. The group of Peirouvi research investigated the differentiation of the hippocampal neural stem/progenitor cells isolated from 3-month-old Wistar rats in response to the embryonic cerebrospinal fluid (eCSF) including E13.5, E17-CSF and the adult cerebrospinal fluid (aCSF), all extracted from rats. In their study they observed that hipp-NSC react differently to the eCSF and aCSF and that the effect depends on the concentration of CSF in the medium. They reported that hipp-NS/PCs were highly neurogenic in response to E13.5 and E17-CSF, but eCSF had no significant effect on astrogliogenesis. Hipp-NS/PCs exposed to aCSF increased GFAP+ expression and decreased MAP2 expressions, which indicates that aCSF promotes differentiation to glial cells (Peirouvi et al., 2015).

In addition, CSF benefits, such as NSC survival enhancement, have been observed *in vivo*. One of the possible administration routes for cell graft is intrathecal (Suzuki et al., 2022). Many studies on NSC transplantation in neurological diseases models used rodent cells and rodent hosts. For example, in the study of Wu and coworkers, rat neurospheres injected into the CSF of a rat with a spinal cord injury migrated into the lesion site and integrated into the spinal cord tissue (Wu et al., 2002). Good survival of NSCs, migration and integration with the injured spinal cord of rat NSCs has been seen also by Bai et al. (2003). There are also studies where human NSCs are transplanted into the rodent models, including transgenic mice, which can mimic the pathological and behavioral mechanisms occurring in neurodegenerative diseases. Such studies are crucial in the case of following the hNSCs reactions and further future transplantations into a human (Willis et al., 2020). However, it seems like there is a lack of studies that would be concentrated on investigating the exact CSF influence in the aspect of the final study outcome.

### 3.2 Effect of CSF on iPSCs-derived NSCs

The multiplicity of ethical issues and concerns regarding the usage of primary NSCs derived from human fetal or embryonic tissues may limit the scope of future research in the field of stem cell-based therapies in neurological diseases. Induced pluripotent stem cells (iPSCs), which are induced from autologous or non-autologous (e.g., homologous/allogenic) cell sources, may be the answer for the future. Their favorable properties can be exploited in many ways, firstly, iPSCs, by the use of commercially available protocols, can be placed in an environment rich in stimulus factors that promote iPSCs’ differentiation into the specified direction such as NSCs, mature neurons or other cells of the nervous system (Galiakberova and Dashinimaev, 2020).

The iPSCs derived-NSCs have been used in Izsak’s group *in vitro* research in 2020. The unique methodological approach allowed the team to obtain iPSCs-derived NSCs cultured as 3D aggregates, which they decided to culture in a healthy adult human cerebrospinal fluid (aCSF) environment. Their results showed that aCSF can be the physiological equivalent of the human healthy brain environment and can enhance the maturation of the functional neuronal network compared to the standard used medium. The Izsak’s team showed that aCSF cells’ incubation may also trigger several processes responsible for neural circuit development in the iPSCs-NSCs 3D model including electrophysiological activity, neuro and astrogenesis, and synapse formation. Interestingly, at the same time, they demonstrated suppression of cell proliferation which may be related to the plethora of factors contained in human cerebrospinal fluid derived from healthy volunteers (Izsak et al., 2020). Such positive effect of aCSF on the neurogenic, astrogenic, proliferation, and synapse formation potential of these cells is related to the one observed by aforementioned groups working on native NSCs.

Due to the lack of relevant research related to the influence of aCSF on iPSCs-derived NSCs, we still have limited information about the behavior and differentiation pattern of iPSCs-derived NSC cells in both *in vitro* and *in vivo* studies.

### 3.3 Effect of CSF on ESCs-derived NSCs

Another approach to studying the effects of cerebrospinal fluid on the therapeutic properties of stem cells, and NSCs in particular, in neurodegenerative diseases may involve the use of NSCs derived from embryonic stem cells (ESCs). ESCs isolated from human embryos are characterized by major ethical concerns, but their superior biological properties related to the ability to pluripotency and differentiation can potentially provide a good source for obtaining NSCs.

The properties of human embryonic stem cells have been employed by Kiiskii et al. group *in vitro* research in which they described the influence of adult healthy human cerebrospinal fluid (aCSF) on hESC-derived neural crest cell properties. Their research revealed that culturing hESCs-derived neural crest cells in the environment of aCSF may change the differentiation potential of NSCs and redirect their expansion toward glial cells at the expense of neuronal differentiation. However, after 4 weeks of hESCs-NSCs cultivation in aCSF the expression of genes related to neural precursors and neurons, astrocytes, and oligodendrocytes phenotypes has been observed by researchers. What is more, the aCSF microenvironment has been revealed to be enriched in FGF2, B-NGF, PDGF, and VEGF-A proteins, but also reduced hESCs-derived NSCs proliferation rate at the same time. Interestingly, despite the glial vs. neuronal differentiation pattern switch and proliferation ability reduction of NSCs, the researchers did not observe changes in neuronal network activity (Kiiski et al., 2013).

In other scientific reports, authors attempted to investigate the behavior of hESC-derived NSC under the alteration of aCSF obtained from neurologically disordered individuals, such as Cristofanilli’s group, whose study used aCSF from volunteers diagnosed with progressive multiple sclerosis disease. This is another confirmation that factors produced during neurological diseases, such as multiple sclerosis can influence NSC survival and therapeutical properties after transplantation. The Cristofanilli et al. group discovered in their *in vitro* studies, that neural progenitor cells (NPCs) differentiated from hESCs, treated with multiple sclerosis patients-derived aCSF may be characterized by reduced proliferation potential with no affection of cells survival. Moreover, researchers found that CSF-treated NPSs significantly upregulate genes associated with differentiation into neurons and oligodendrocytes, but not astrocytes. Acquired outcome has been verified, by other techniques analysis, in which the enhanced astrocytic differentiation potential was not confirmed. The authors suggested that soluble factors dispensed in multiple sclerosis patients-derived CSF may play an important role during the redirection of NPCs from proliferation to differentiation state in order to rebuild the pool of the lost neural cells during the course of the disease (Cristofanilli et al., 2013).

To investigate how aCSF affects the functional activities of nervous system cells, the group of Sumitha, used hESCs-derived motor neurons that were incubated in aCSF derived from sporadic Amyotrophic Lateral Sclerosis (ALS). Authors observed a number of toxic effects of ALS-derived aCSF on ESC-derived motor neurons and noticed early signs of neurodegeneration such as lower viability, increased apoptotic proteins, impaired mitochondrial complex activities, hyperexcitability, organelles alterations and downregulation of BDNF expression (Sumitha et al., 2019). Presented studies are in accordance with the Brauer group findings, in which they also demonstrated the harmful effect of ALS-derived aCSF on iPSCs-derived motor neurons (Bräuer et al., 2020).

All of the presented studies lead us to the belief that the origin of aCSF itself plays an important role in terms of modifying ESC phenotype and function. Differences in composition of CSF might determine a multitude of processes taking place in cells, including neural differentiation or proliferation.

### 3.4 Effect of CSF on other cell types

In this review, we also decided to analyze the impact of CSF on other stem cells due to the large data provided. As the derivation of native NSCs from the primary tissues can be problematic and invasive, and the performed procedure can raise ethical issues, the researchers try to find other effective sources of these cells. So far, most studies on CSF have been performed on mesenchymal stem/stromal cells (MSCs). Using MSCs in regenerative medicine seems to bring many benefits. Autologous MSCs can be easily isolated, cultured, and directed into cells expressing neural markers and secreting altered, responsible for neuroprotection cytokines, chemokines, and morphogenes (Nivet et al., 2011; Tang et al., 2017; Zhang et al., 2017; Kaminska et al., 2022). However, it should be emphasized that mostly, their neurogenic abilities are presented in the increased expression of neural markers, which could be linked not with actual differentiation. For example, it has been suggested, that βIII-tubulin expression can be induced due to phenotypic changes in passaging and may be related to reorganization in the cytoskeleton (Słysz et al., 2022), and was proposed to be a common feature of MSCs (Drela et al., 2014).

It has been observed that short-term culture in artificial CSF (art-CSF) did not affect the morphology of adipose-derived mesenchymal stem/stromal cells (AD-MSCs) (Krull et al., 2021), however, after 9 days of such culture in adult human CSF (derived from benign intracranial hypertension (BIH) patient), morphological changes toward neural-like cells were observed (Elgamal et al., 2019). Furthermore, culture in adult human CSF increased the proliferation and viability of AD-MSCs (Zhu et al., 2015), whereas the culture in art-CSF decreased both features (Zhu et al., 2015; Krull et al., 2021). Regarding adult human CSF, it was shown to elevate the gene expression of migratory marker CXCR4 (C-X-C Motif Chemokine Receptor 4) (Zhu et al., 2015) and Nestin, with no changes of MAP2 (microtubule-associated protein 2) (Elgamal et al., 2019). Interestingly, the use of a lower concentration of adult human CSF resulted in a decreased gene expression of GFAP (glial fibrillary acidic protein) while a higher concentration increased its expression (Elgamal et al., 2019). AD-MSCs cultured in art-CSF did not express the pluripotent genes and Ki67 (a marker of proliferation) but expressed PCNA (proliferating cell nuclear antigen) (Krull et al., 2021). In another study, adult human CSF increased the number of Ki67^+^ cells, while art-CSF decreased the amount of Ki67^+^ cells (Zhu et al., 2015). These studies showed that art-CSF should not be treated like a substitute for adult human CSF in studying the effects of CSF on MSCs.

Moreover, in the study where AD-MSCs were administered via intrathecal injection, a group of Kuzma-Kozakiewicz observed that TNF-alpha (tumor necrosis factor alpha) level was decreased while FGF basic (basic fibroblast growth factor), IL-6 (interleukin 6) and MMP-6 (Matrix Metallopeptidase 6) levels were increased in CSF after the treatment with AD-MSCs over patients with amyotrophic lateral sclerosis (ALS) (Kuzma-Kozakiewicz et al., 2018). Another group pointed out that the treatment of AD-MSCs in Autoimmune Refractory Epilepsy resulted in increased angiogenin, CXCL12/SF1alfa I and IL-10 (interleukin 10) and decreased osteopontin levels (Szczepanik et al., 2020).

Culturing bone marrow mesenchymal stem/stromal cells (BM-MSCs) in CSF resulted in morphological changes to present astrocyte dendrite and axon-like cells [human CSF and human BM-MSCs; (Ye et al., 2011; Ge et al., 2015)] neurite length [rat CSF and murine BM-MSCs; (Shokohi et al., 2017)]; long spindle-shaped cells [rat CSF and rat BM-MSCs; (Mafikandi et al., 2019)]; mature neurons and dendrite-like cells presenting Nissl bodies [rabbit CSF and rabbit BM-MSCs; (Otify et al., 2014)]. Moreover, CSF from newborn rats of healthy mothers (N-CSF, normal-CSF) has been shown to undergo more rapid morphological change into long spindle-shaped cells than CSF from newborn rats of mothers with hypothyroidism (HTH-CSF, hypothyroidism-cerebrospinal fluid) (Mafikandi et al., 2019). The presence of CSF (human and rat) increased protein expression of β-III-tubulin in human (Ye et al., 2011; Ge et al., 2015) and murine BM-MSCs (Shokohi et al., 2017). Moreover, an increased GFAP expression has been observed [in human and rabbit CSF; (Ye et al., 2011; Otify et al., 2014; Ge et al., 2015)]. Different effect of CSF on stem cells has been also observed depending on their origin. Shokohi et al. (2017) observed that CSF affected the expression of marker MAP2 (increase) in murine BM-MSCs, but did not affect MSCs derived from dental pulp (Haratizadeh et al., 2017). In addition, rat CSF increased the expression of the neural marker Nestin in dental pulp MSCs (Haratizadeh et al., 2017) and rat BM-MSCs (Mafikandi et al., 2019). Rat BM-MSCs maintained higher viability in N-CSF than in HTH-CSF (Mafikandi et al., 2019). In addition, viability was increased when the concentration of rat CSF in the medium was increased to 10%. However, viability was reduced at 20% rat CSF in the medium (Haratizadeh et al., 2017). The culture of human BM-MSCs in aCSF showed they secreted BDNF (brain-derived neurotrophic factor), CNTF (ciliary neurotrophic factor), TGF-β (transforming growth factor β) and possess antioxidant properties. Interestingly, aCSF used for BM-MSCs culture increased the viability of PC12 and SH-SY5Y (Valitsky et al., 2019). BM-MSCs are also used in clinical trials of neurodegenerative diseases using intrathecal injection. The administration of BM-MSCs to patients with active progressive multiple sclerosis resulted in NF-L (neurofilament light chains) and CXCL13 (chemokine receptor) decrease in CSF (Petrou et al., 2022). In patients with ALS, increased levels of TGF-β1-3, IL-6, IL-10 (Oh et al., 2015, 2018), TGF-β2, TGF-β3 (Oh et al., 2015), VEGF (vascular endothelial growth factor), HGF (hepatocyte growth factor), LIF (leukemia inhibitory factor) (Berry et al., 2019) and reduced levels of MCP-1 (monocyte chemoattractant protein-1) (Oh et al., 2018; Berry et al., 2019), SDF-1 (stromal cell-derived factor-1), CHIT-1 (chitotriosidase-1) (Berry et al., 2019) were observed.

It has been proven that CSF also has a considerable impact on WJ-MSCs (mesenchymal stem cells derived from Wharton’s Jelly of umbilical cord). When cultured in the presence of CSF (100%), changes in cell morphology and proliferation rate have been observed over time. While WJ-MSCs usually represent a typical fibroblast-like morphology, in the presence of CSF the cells became elongated and formed axon-like protrusions. WJ-MSCs exhibited a high proliferation rate for the first 3 days of *in vitro* culture, which then slowed down between 3 and 5 days of culture. The cells stopped proliferating after 5 days of culture (Sypecka et al., 2022). In the aforementioned study, the authors also investigated whether WJ-MSCs cultured in CSF undergo neural differentiation. It turned out that WJ-MSCs cultured in CSF expressed higher levels of specific neural markers such as: Nestin, β-III-tubulin, S-100-β, GFAP, and doublecortin than those cultured in standard culture medium. Moreover, RT-qPCR analysis revealed that WJ-MSCs cultured in CSF expressed higher levels of MAP2 and NeuN than those cultured in standard culture medium. However, when it comes to NG2, the expression level of this gene was decreased in WJ-MSCs cultured in CSF. Based on these results, the authors suggest that WJ-MSCs undergo neural differentiation in the presence of CSF (Ge et al., 2015). Other studies report that even a small addition of CSF (10 μL/2 mL of DMEM or 100–200 μL/mL of DMEM, without platelet lysate) to the culture medium triggers changes in WJ-MSCs’ phenotype. Changes in cell morphology were observed as before – after 3 days of culture they became irregular, and many were triangle-shaped; after 7 days of culture the cells formed characteristic, axon-like protrusions. It was also observed that cells cultured in standard culture medium did not express GFAP, MAP2 and β-III-tubulin. However, the addition of CSF to the medium triggered the expression of these markers (Farivar et al., 2015; Pellegrini et al., 2020).

The aforementioned studies reveal a crucial impact of CSF on MSC morphology, differentiation, and protein expression. Notably, CSF from different sources, such as human, rat, mouse, and rabbit, exhibits various effects on MSC behavior, underlining the complexity of their interactions. In addition, the results obtained with the use of artificial vs. healthy vs. diseased patient-derived CSF differ from each other.

The influence of CSF was also studied on other types of stem cells. For example, it has been investigated whether CSF can stimulate the therapeutic potential of multipotent stem cells residing in the bulge of hair follicles – epidermal neural crest stem cells (EPI-NCSCs) for their further use in neurodegenerative disorders treatment (Pandamooz et al., 2013). To do so, a group of Pandamooz investigated the fate of mouse EPI-NCSs cultured in adult rat CSF. The researchers observed a decrease in cell proliferation and differentiation inhibition in such conditions, which in their opinion could be beneficial for transplanted cells because of a possible direct differentiation as a response to the signals of the target injured site, making CSF a great route of cell administration to CNS. However, it is noteworthy that the used CSF was obtained from healthy donors of different species (Wistar rats) than the used cells (mice). The same group has recently published a letter suggesting the potential of using neural crest-derived stem cells from hair follicles in Parkinson’s disease treatment, thanks to their ability to generate dopaminergic neurons as a response to eCSF presence (Pandamooz et al., 2022). A similar effect on neuronal differentiation was presented in a study in which human dental pulp stem cells (hDPSCs) were investigated (Goudarzi et al., 2020). This research showed, that CSF isolated from the *cisterna magna* of 19-day-old Wistar rat embryos added to the culture even for 2 days can induce final differentiation to neuron-like cells from hDPSCs, resulting in expression of Nestin, MAP2 and the presence of Nissl bodies in the cytoplasm (Table 2).

**Table 2:** Comparison of studies of CSF effect on NSCs.

Primitive cell type		CSF source	% of CSF in the medium	CSF-induced effect	CSF-induced cell type	Proposed mechanism of action	*In vitro*/*in vivo* studies	References
NSCs	Mesencephalic NSCs from E14.5 rat embryonic brain	Adult human leptomeningeal CSF		NSC survival enhancement; glial differentiation stimulation; neurogenesis inhibition; proliferative and migratory potential decrease	Promoted glial differentiation; reduced proliferation	–	*in vitro*	Buddensiek et al. (2009)
	Adult human hippocampal tissue obtained from routine epilepsy surgery procedures	Adult human leptomeningeal CSF		NSC survival enhancement; glial differentiation stimulation; proliferative potential decrease	GFAP+ cells increase	BMP4 was shown to induce neuronal differentiation of NSCs by activating the ERK and inhibiting the GSK3b pathway. BMP effects could be blocked by BMP inhibitor - noggin. However, in the article authors did not find any inhibiting effects of noggin.	*in vitro*	Buddensiek et al. (2010)
	Rat sympathetic neurons	Adult bovine CSF		Increase in dendritic growth		BMP-7 is known to induce dendritic growth. To examine whether the dendritic growth induced by CSF was due to BMPs, cultures were treated with follistatin, a protein that binds and sequesters activin and some BMPs. Follistatin caused a significant reduction in CSF-induced dendritic growth, as did noggin.	*in vitro*	Dattatreyamurty et al. (2001)
	Primary OPC cultures isolated from naïve P0 to P1 neonatal C57/BL6 murine cortices	Adult human CSF from relapsing and progressive multiple sclerosis patients	10–20%	CSF from pMS impedes OPC differentiation to mature oligodendrocytes; OPCs exposed to CSF from rMS were more morphologically mature compared with CSF from pMS; enhanced immune activity	Mature O1+ oligodendrocytes	–	*in vitro*	Zveik et al. (2022)
	Neurospheres obtained from NSPCs from the ventricular and subventricular zones (VZ/SVZ) of PN1 normal, non-hydrocephalic HTx rats.	CSF collected from nHTx and hyHTx rats	5% CSF from nHTx rats or 5% CSF from hyHTx	CSF enhances the differentiation of NEs into neurons and astrocytes	Nestin+ (65% of the cells); GFAP+(22%) βIII-tub+ (20%)/neurons, glial cells and ependymal cells		*in vitro*	Henzi et al. (2018)
	Mesencephalic NSCs from E14 rat fetuses	Human CSF	1 mL CSF	No neuronal differentiation, no NEs; CSF cannot support newborn neurons to survive	GFAP+/glial cells		*in vitro*	Ma T. et al. (2013)
	NSCs obtained from rat embryonic day 15 (E15) fetal brain	Human CSF from patients with subarachnoid hemorrhage (SAH)	0.5%	Enhancement of neurogenesis and proliferative potential of NSCs			*in vitro*	Chen et al. (2018)
	Adult rat hippocampal neural stem/progenitor cells (hipp-NS/PCs)	Rat embryonic CSF (eCSF) and rat adult CSF (a-CSF)	15% or 20%	eCSF: enhancement of neuronal differentiation; no strong effect on astrogliogenesis aCSF: enhancement of Gliogenesis; neurogenesis inhibition	eCSF: MAP2+ neurons aCSF: GFAP+		*in vitro*	Peirouvi et al. (2015)
ESCs	Human embryonic stem cells-derived NSCs	Human, healthy adult patients	100%	Glial cells	Promoted glial differentiation; reduced proliferation		*in vitro*	Kiiski et al. (2013)
	Human embryonic stem cells-derived NPCs	Human, multiple sclerosis patients	5%	Neurons and oligodendrocytes	Reduced proliferation; neuronal and oligodendrocytic differentiation enhancement; survival maintenance		*in vitro*	Cristofanilli et al. (2013)
	Human embryonic stem cells-derived motor neurons	Amyotrophic lateral sclerosis patients; intracranial hypertension patients	10%	Not applicable	Induced apoptosis; reduced viability; organelle alterations; decrease of BDNF expression		*in vitro*	Sumitha et al. (2019)
Other stem cells	Human AD-MSCs	31-year-old woman with BIH	0.5%	Neural-like cells, increased proliferation rate decreased gene expression of GFAP and Nestin	Inhibited neural differentiation		*in vitro*	Elgamal et al. (2019)
		31-year-old woman with BIH	2.5%	Neural-like cells, increased proliferation rate decreased gene expression of GFAP and Nestin	Inhibited neural differentiation		*in vitro*	
		31-year-old woman with BIH	5%	Neural-like cells, increased proliferation rate decreased gene expression of GFAP and Nestin	Inhibited neural differentiation		*in vitro*	
31-year-old woman with BIH		10%	Neural-like cells, increased proliferation rate increased gene expression of GFAP decreased gene expression of Nestin	Inhibited neural differentiation, glial differentiation		*in vitro*		
Human AD-MSC	Human	100%	No changes in morphology	Fibroblast-like		*in vitro*	Krull et al. (2021)
	Artificial	100%	Decreased metabolic activity, proliferation, and viability no expression of pluripotent gene, Ki67	–		*in vitro*	
Human AD-MSC	Human	25%	Low proliferation low number of Ki67 high % of apoptosis	–	Neutralization of IGF-1 in human CSF decreased migration and proliferation cells compared to non-neutralized human CSF, in contrast to the cells cultured in standard medium; the rate of apoptosis in neutralized human CSF was decreased compared to the standard medium but increased in human CSF	*in vitro*	Zhu et al. (2015)
	Artificial	25%	High proliferation high number of Ki67 Low % of apoptosis High gene expression of CXCR4	–		*in vitro*		
BM-MSCs	Artificial	–	Secrete BDNF, CNTF, TGF-β, and anti-oxidant capacity	–		*in vitro*	Valitsky et al. (2019)	
Human dental pulp MSCs	Newborn rat race Sprague–Dawley	10%	Neuron-like cells high level of markers: nestin and GFAP	Neural progenitor, astrocyte cells		*in vitro*	Haratizadeh et al. (2017)	
Human BM-MSCs	Healthy human	–	Increased markers of β-III-tubulin and GFAP	Astrocyte dendrite, axon-like cells		*in vitro*	Ge et al. (2015)	
Rat BM-MSCs	Healthy newborn rats	5%	High gene expression of nestin Low gene expression of NEORD-1	–		*in vitro*	Mafikandi et al. (2019)	
Rat BM-MSCs	Hypothyroid newborn rats	5%	High gene expression of nestin, NEORD-1, and NEUN	–		*in vitro*	Mafikandi et al. (2019)	
Murine BM-MSCs	Rat fetuses	10%	Longer neurite length			*in vitro*	Shokohi et al. (2017)	
Human BM-MSCs	Healthy human	10 μl every day for 7 days	Increased markers of β-III-tubulin and GFAP	Astrocyte dendrite, axon-like cells		*in vitro*	Ye et al. (2011)	
Rabbit BM-MSCs	Rabbit	10 μl every day for 9 days	Appearance of Nissl bodies, glycogen granules	Mature neurons and dendrite-like cells, astrocyte		*in vitro*	Otify et al. (2014)	
WJ-MSCs	Healthy patients	100%	Neural-like morphology, expression of neural markers and genes (Nestin, β-III-tubulin, S-100-β, GFAP, doublecortin, NeuN, MAP2)	Neural		*in vitro*	Sypecka et al. (2022)	
Umbilical cord blood-derived MSCs	Healthy patients	10 μL per 2 mL of medium	Neural-like morphology, expression of GFAP, β-III-tubulin	Neural		*in vitro*	Ge et al. (2015)	
WJ-MSCs	Healthy patients (children)	100 μL or 200 μL per mL of medium	Expression of Nestin, GFAP, MAP2	Neural		*in vitro*	Farivar et al. (2015)	
EPI-NCSCs isolated from 3-week-old NMRI mice	CSF was collected from the cisterna magna (CM) of Wistar rats	10%	CSF maintained the expression of nestin, β–tubulin ІІІ (early neuronal marker), and glial fibrillary acidic protein (GFAP, glia marker)	Decreased cell proliferation rate, CSF does not promote cell differentiation toward any specific destiny		*in vitro*	Pandamooz et al. (2013)
	Human dental pulp stem cells (hDPSCs) were	From the Cisterna magna of 19-day-old Wistar rat embryos	5%	Expression of a neural progenitor marker (Nestin) and a mature neural marker (MAP2) Nissl bodies in cell cytoplasm	Promoted neural and neuronal differentiation		*in vitro*	Goudarzi et al. (2020)

## 4 Potential roles of CSF in future cell therapy development for neurological disorders

NSCs play a pivotal role in the formation and maintenance of the entire nervous system. Their defects or damage lead to serious neurological consequences, thus, more and more studies are performed in order to understand the mechanisms involved in CNS dysfunctions. Along with the growing number of studies, there is a rising discussion in the aspect of some even contradictory findings. Previously, we stressed the importance of interpreting the results obtained after performing different culture conditions on the same cell type, as well as misleading findings using the same conditions regarding different species origin (Radoszkiewicz et al., 2023a,b). We pointed out that in many cases the protocols standardized on stem cells from animal models cannot be directly applied to human stem cells. We should also keep in mind that animal models of neurological disorders do not fully mimic the ones of human brain, as there are significant developmental differences between the species.

**Figure 2:**
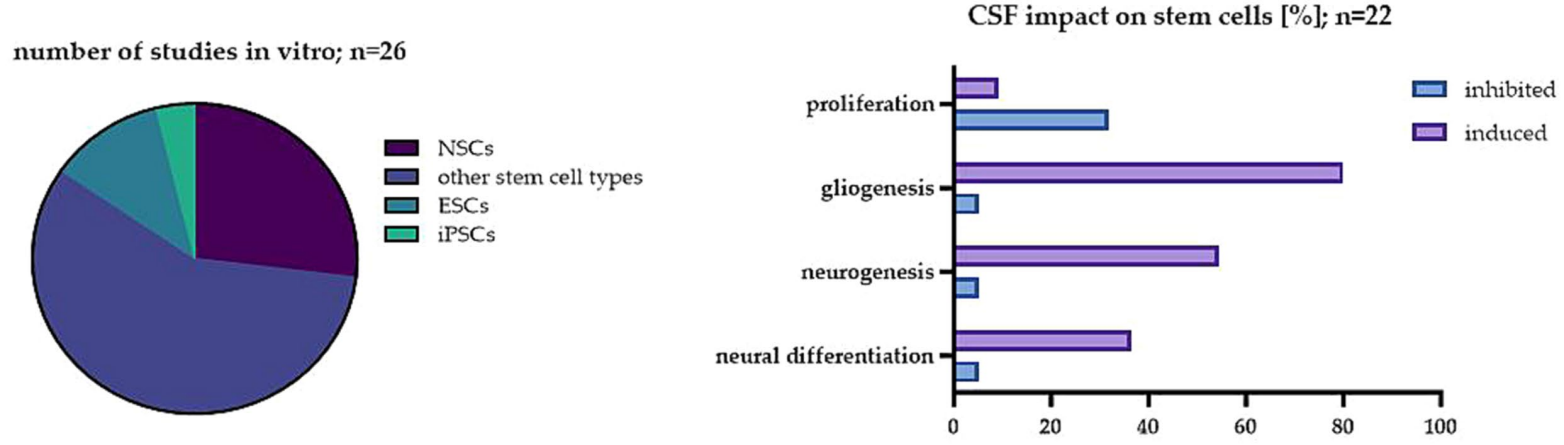
The impact of CSF on stem cells in the investigated studies. On the left: The percentage of studies analyzed *in vitro* in regard to different cell types; on the right: The most common effect of CSF on stem cells in the analyzed studies *in vitro*.

In this paper, we wanted to emphasize the significance of another factor which is still not well-studied *in vitro* and in our opinion, could be critical for striking the right balance between interpreting preclinical and clinical results. Nowadays, CSF is yet considered as a fluid with not only basic mechanical and chemical properties. Its crucial roles seem to change, starting from the complicated processes of neural development up to adulthood. A variety of complex changes in CSF occur in pathologies of neurological disorders. CSF, by transporting several necessary nutrients, hormones, and other factors around the CNS, provides a kind of highway for intricate cell signaling pathways that guarantee the right brain homeostasis. Thus, it is a perfect model to study endogenous as well as exogenous NSC behavior. Here, we analyzed the effect of CSF *in vitro* on different stem cell populations. In 26 of the analyzed studies, the significant influence of CSF on stem cell proliferation, differentiation and survival has been described (Figure 2).

The presented studies have shown that CSF has a great impact on the proliferation of not only native NSCs but also other stem cell populations. This effect is likely due to the presence of specific signaling molecules in the CSF that regulate cell growth and differentiation. Interestingly, the effect depends on the origin of the CSF. The results obtained with the use of artificial CSF differ from the ones obtained with human origin treatment, thus, such exchange is not recommended. Moreover, a significant difference between embryonic CSF and adult CSF has also been shown. It has been already proposed by embryonic CSF studies that diffusible factors in CSF can regulate neuroepithelial stem cell fate, influencing brain development *in vivo* (Gato et al., 2005). While the exact mechanism of its impact on neuroectodermal cells remains unclear, components like proteins, particles, amino acids, and FGF2 were seen to be implicated (Martín et al., 2006; Bachy et al., 2008; Huttner et al., 2008). Despite the complexity of embryonic CSF’s composition compared to adult CSF, it retains the ability to influence the behavior of adult neural stem cells (NSCs) in the brain (Buddensiek et al., 2010). In the NSC studies, eCSF promoted neuronal differentiation, while adult CSF stimulated the cells into glial differentiation. It is worth noting that most of the analyzed studies were performed using adult CSF. Overall, these studies also showed that the proliferation capacity of stem cells seems to be lowered in the CSF obtained from healthy donors. One possible reason for such an inhibitory effect is that CSF contains factors that promote the differentiation of stem cells into specific types of cells, which in this case was mostly into glial cells. However, the CSF from patients with diseases like BIH or SAH enhanced the proliferation of NSCs. The possible explanation of this effect is that CSF from patients with these conditions contains higher levels of growth factors and cytokines that promote cell proliferation, such as those previously shown to be present during regeneration after brain injury-FGF, NGF, TGF-β, GDNF, BDNF, VEGF, which possess neuroprotective properties, improve the survival and proliferation rate of neurons (Lehtinen et al., 2011). It has been shown that CSF from patients with SAH contains elevated levels of VEGF or BDNF which are known to stimulate the growth of blood vessels, promote tissue repair and play a key role in post-SAH proliferation of NSCs (Sgubin et al., 2007; Chen et al., 2013, 2018).

In addition, in most of the described studies, the presence of CSF improved the differentiation into glial cells. The exact mechanisms of action are, however, not well-described. Buddensiek’s group suggested that the enhanced differentiation is a result of BMP presence in CSF expressed by the choroid plexus (Buddensiek et al., 2010). It is not confirmed whether the choroid plexus expresses BMPs like BMP2, BMP3, BMP4, BMP5 and BMP6, however, it is known that BMP4 stimulates the differentiation via ERK pathway activation and GSK3β inhibition (Dattatreyamurty et al., 2001; Moon et al., 2009; Buddensiek et al., 2010; Jensen et al., 2021). Dattatreyamurty and coworkers also investigated BMP presence in CSF, by examination of BMP-7’s known ability to induce dendritic growth in rat sympathetic neurons. To confirm that the dendritic growth induced by CSF was due to BMP-7, cultures were treated with BMP inhibitors – follistatin and noggin. Follistatin caused a significant reduction in CSF-induced dendritic growth, as did noggin. In addition, dendritic growth induced by bovine CSF was inhibited by function-blocking antibodies against BMP-7, i.e., 72% inhibition with 12G3 and 40% inhibition with 1B12. These observations suggest that a substantial portion of the dendrite-promoting activity in CSF is due to BMP-7 (Dattatreyamurty et al., 2001). In the study of the Zhu group, a noteworthy positive influence exerted by insulin-like growth factor-1 (IGF-1) was found in human CSF on the migration capacity and C-X-C chemokine receptor type 4 (CXCR4) expression in both human exogenous primary amniotic MSCs and fetal neural progenitor cells. The authors also confirmed the impact of CSF on the proliferation, migration, and viability of these stem cell types (Zhu et al., 2015). It has been also discovered that embryonic CSF, particularly CSF-insulin-like growth factor 2 (IGF-2), plays a vital role in providing factors that stimulate the growth and survival of the developing rodent cortex (Lehtinen et al., 2011). Moreover, in the developing chick brain, it has been seen that CSF contributes to the retention of midbrain markers such as Otx2 and Fgf8, while FGF2 in CSF seems to promote precursor proliferation (Parada et al., 2005b; Martín et al., 2006). In addition, within the mouse cerebellum, CSF-distributed Sonic Hedgehog (Shh) has been proposed to stimulate the proliferation of cerebellar granule neuron precursors (Huang et al., 2010). It has been also shown that CSF contains high levels of other cytokines, such as leukemia inhibitory factor (LIF) and ciliary neurotrophic factor (CNTF), which promote the differentiation of NSCs into astrocytes (Nakanishi et al., 2007). Such effect could also be caused by TGF-β, which is known to play a key role in brain development, brain homeostasis during adulthood and neurological pathologies. TGF-β-1 signaling is one of the major pathways that regulate gliogenesis (Stipursky and Gomes, 2007; Diniz et al., 2018). What is more, the same factor seems to be responsible for neurogenesis inhibition, which also occurred in the analyzed studies. It has been presented as a molecule modulating the neural stem and progenitor cell proliferation in the CNS (Wachs et al., 2006). In mice hippocampal neurons, TGF-β induced cell cycle exit (Vogel et al., 2010). Moreover, it was shown to induce neurite growth, which was also observed in some of the investigated studies (Knöferle et al., 2010; Hiew et al., 2021).

So far, different effects of CSF on NSCs, even the ones of the same origin, have been described. The reasons for such discrepancies between groups can be traced not only to the different sources of the NSCs, but also to the amount of CSF used in cell culture (starting from 0.5% up to 100% in the culture medium), or finally, the biodiversity and origin of the CSF itself. However, those findings provide direct evidence that healthy cerebrospinal fluid, circulating through different parts of the CNS, may provide a beneficial environment for the administrated NSCs due to the presence of potentially stimulatory factors for neural lineage differentiation at the expense of reducing proliferation.

In summary, the study analysis confirmed that further experiments to examine the fate of NSCs under specific influences of CSF are required. To understand the effects of CSF on these cells, it is important to further study the complex interactions between NSCs and CSF, including various growth factors, signaling molecules, and other factors present. Knowing the conditions in which CSF could support NSCs and promote regeneration and restoration could allow to improve targeted cellular therapy in CNS disorders. It’s important to note that the effects of CSF on neurogenesis are complex and may depend on several different factors, including the age of the individual, the specific components of the CSF, and the specific brain region being studied. The CSF seems to be a potential vehicle for volume transmission of growth factors, implanted neural stem cells, and chemokines. As such, the CSF as a therapeutic vehicle to promote CNS homeostasis was described in animal models many times-however, the papers analyzing the effects of unaltered or naturally altered CSF on neurogenesis are very few. Thus, further research is needed to fully understand the relationship between CSF and neurogenesis.

Summary notes:

- CSF appears to provide a supportive microenvironment for NSCs, influencing their survival, and differentiation.
- Adult CSF environment influences iPSCs-derived NSCs, directing them toward neurogenesis or gliogenesis while suppressing proliferation. It promotes synapse formation, neurite outgrowth, and activation of neuronal circuits in these cells.
- ESCs-derived NSCs cultured in aCSF were seen to have changes in their differentiation potential, leading to the formation of glia, neural precursors, neurons, astrocytes, or oligodendrocytes. Additionally, there was an observed decrease in the proliferation rate of ESCs-derived NSCs during aCSF culture.
- ALS-derived aCSF negatively affects ESCs-derived NSCs. Harmful effects include lower viability, vacuolization, and the induction of apoptosis in ESCs-derived NSCs when exposed to ALS-derived aCSF.
- Irrespectively of the source, CSF affected MSCs properties such as morphology, proliferation, viability, neural gene expression and secretome which varied between the studies.
- The effects of CSF vary depending on its origin, with differences observed between artificial CSF and human-origin CSF.
- The effects of CSF vary based on factors such as cell sources, CSF concentration, and CSF origin.
- Adult CSF from healthy donors may inhibit stem cell proliferation, potentially due to factors promoting differentiation into specific cell types, mainly glial cells.
- Healthy CSF may provide a beneficial environment for NSCs, supporting neural lineage differentiation at the expense of reduced proliferation.
- Limited research on the effects of unaltered or naturally altered CSF on neurogenesis emphasizes the need for additional studies.
- This review underscores the importance of further research to comprehend the relationship between CSF and neurogenesis, with potential implications for improving targeted cellular therapy in CNS disorders.

## Author contributions

KR: Conceptualization, Formal analysis, Writing – original draft. AB: Formal analysis, Writing – original draft. MC: Writing – original draft. PR: Writing – original draft. MS: Writing – original draft. IZ-K: Writing – original draft. AS: Supervision, Writing – review & editing.

## Figures and Tables

**Table:** 

NSCs	neural stem cells
AD-MSCs	adipose tissue – mesenchymal stem/stromal cells
CSF	cerebrospinal fluid
aCSF	adult cerebrospinal fluid
art-CSF	artificial cerebrospinal fluid
eCSF	embryonic cerebrospinal fluid
CNS	central nervous system
BIH	benign intracranial hypertension
PRP	platelet-rich plasma
MAP2	Microtubule-associated protein 2
GFAP	Glial fibrillary acidic protein
KI67	marker of proliferation Ki67
PPARG	peroxisome proliferator activated gamma
hDPSCs	human dental pulp stem cells
EPI-NCSCs	epidermal neural crest stem cells
SOX9	SRY-Box transcription factor 9
ALPL	alkaline phosphatase
RUNX2	runt-related transcription factor 2
COL21A1	collagen type XXI alpha 1 chain
MGP	matrix glia protein
MSC	mesenchymal stem/stromal cells
OGN	osteoglycin
PODN	podocan
CXCR4	C-X-C Motif Chemokine Receptor 4
TNF-alpha	tumor necrosis factor alpha
FGF basic	basic fibroblast growth factor
IL-6	interleukin 6
MMP-6	Matrix Metallopeptidase 6
ALS	amyotrophic lateral sclerosis
IL-10	interleukin 10
BM-MSCs	bone marrow – mesenchymal stem/stromal cells
BDNF	brain-derived neurotrophic factor
CNTF	ciliary neurotrophic factor
TGF-β	transforming growth factor β
N-CSF	normal CSF
Shh	Sonic Hedgehog
HTH-CSF	hypothyroidism-cerebrospinal fluid
NEURD-1	neurogenic differentiation 1
NeuN	neuronal nuclei
NF-L	neurofilament light chains
CXCL13	chemokine receptor
VEGF	vascular endothelial growth factor
HGF	hepatocyte growth factor
LIF	leukemia inhibitory factor
MCP-1	monocyte chemoattractant protein-1
SDF-1	stromal cell-derived factor-1
CHIT-1	chitotriosidase-1
PCNA	Proliferating cell nuclear antigen
